# Visualized analysis of trends and hotspots in global oral microbiome research: A bibliometric study

**DOI:** 10.1002/mco2.47

**Published:** 2020-12-10

**Authors:** Ga Liao, Jinyun Wu, Xian Peng, Yuqing Li, Li Tang, Xin Xu, Dongmei Deng, Xuedong Zhou

**Affiliations:** ^1^ State Key Laboratory of Oral Diseases National Clinical Research Center for Oral Diseases, West China Hospital of Stomatology Sichuan University Chengdu China; ^2^ Department of Cariology and Endodontics, West China Hospital of Stomatology Sichuan University Chengdu China; ^3^ Medical Big Data Center Sichuan University Chengdu China; ^4^ Department of Information Management Department of Stomatology Informatics, West China Hospital of Stomatology Sichuan University Chengdu China; ^5^ Department of Preventive Dentistry, Academic Center for Dentistry Amsterdam (ACTA) University of Amsterdam and VU University Amsterdam Amsterdam Netherlands

**Keywords:** bibliometric, CiteSpace, knowledge map, oral microbiome, WoSCC

## Abstract

The oral microbiome contains numerous bacteria, which directly or indirectly participate in various human functions and continuously exchange signals and substances with the human body, significantly affecting human life cycle, health, and disease. This study aimed to conduct bibliometric studies on the scientific outputs of global oral microbiome research by Citespace software. The data were obtained from the Thomson Reuters' Web of Science Core Collection (WoSCC), from the first relevant literature published until December 31st, 2019, and a total of 2225 articles and reviews were identified. The top country and institutions are the United States and Harvard University. Keywords analysis showed that periodontal disease, oral microbes, and dental plaque are research hotspots. The burst word analysis indicates that early childhood caries, squamous cell carcinoma, gut microbiome, Helicobacter pylori, Candida albicans, and dysbiosis are likely to become the research hotspots of the next era. We also recommend the use of knowledge mapping methods to track specific knowledge areas efficiently and objectively regularly, which can accurately identify hotspots and frontiers and provide valuable information for practitioners in the field, including related scientists, students, journals, and editors.

## Graphical abstract

This study aimed to study the scientific outputs of the global oral microbiome research. The data were obtained from the WoSCC, from the earliest literature to December 31st, 2019. Interesting insights are discovered, such as research hotspots and frontiers. We recommend using bibliometric methodology to dynamically monitor a specific field to achieve comprehensive understanding and trend on a regular basis.

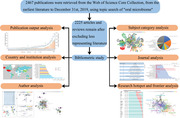

## 1 INTRODUCTION

The term “human microbiome” refers to trillions of commensal, beneficial, and pathogenic bacteria that occupy the human body.^1^ It is often presented as common knowledge that, human microbiome outnumbers human cells by at least 10‐fold, however, the ratio between them may be closer to 1:1 according to the recent studies,^2^ and the interactions between the microbiome and the host factors ultimately mediate the transition from health to disease. Hence, the human microbiome has been recognized as “supraorganisms” of the human body.^3^

As one of the most clinically relevant microbial habitats, the oral cavity is colonized by an extremely diverse oral microbiome, including bacteria, archaea, fungi, and viruses.^4^ Current technology has identified over 1000 different microorganisms, including 700 bacterial species, in the oral cavity.^5^, ^6^ Our group has established an oral microbiome sample bank of the Chinese population, which has laid a good foundation for future research.^7^ Accumulating data have shown that the oral microbiome not only contributes to the development of oral diseases such as dental caries and periodontal diseases,^8^ but also involves in systemic diseases such as diabetes, cardiovascular diseases, rheumatoid arthritis, preterm birth, respiratory diseases, colorectal cancer, inflammatory bowel diseases, and Alzheimer's disease, etc.^9^, ^10^, ^11^ More importantly, a robust interplay between oral microbiome with the gastrointestinal tract has been recently well documented, particularly concerning the role of oral anaerobes in the development and progression of colorectal carcinoma.^10^, ^12^, ^13^, ^14^, ^15^

The early scientific investigations on oral microbial communities can be traced back to around 1680 when van Leeuwenhoek (1632‐1723) observed and described several rod‐ and sphere‐shaped microorganisms in the tartar from his teeth with his handmade microscope.^16^ This primordial observation signaled the complexity of the oral microbial community. In the late 19th century, WD Miller, who is generally recognized as the father of modern oral microbiology, described the critical role of oral microbes in the devolvement of dental caries.^17^ Miller is also the first person who proposed the focal infection theory, which indicates the relationship between oral microorganisms and the development of a variety of diseases such as brain abscesses, pulmonary diseases, and gastric problems. However, Miller failed to recognize the vital role of dental plaque, which is currently known as a typical form of oral biofilm, in the development of dental caries. GV Black, known as one of the founders of modern dentistry in the United States, proposed that oral microorganisms exist in the oral cavity is a complex gelatinous form,^18^ which was defined and characterized as dental plaque by JL Williams,^19^ an American pathologist and prosthodontist in 1897. In 1924, JK Clarke described *Streptococcus mutans* as an etiological factor of dental caries,^20^ and the cariogenic role of this bacterium was further confirmed by a series of animal studies carried out in the mid‐20th century.^21^, ^22^ As guided by the theory that dental caries can be caused by a specific pathogen, isolation, and characterization of individual bacteria associated with dental caries, and the identification of specific virulent factors that could be exploited as a potential target for caries control were well documented in the oral microbiology literature in the 1980s and 1990s.

The definition of “biofilm” by Bill Costerton in 1978 revealed the complexity of microbial consortium and the robust interactions between microbes and the host,^23^ leading to holistic thinking on oral microbial community.^24^ The introduction of 16S rRNA sequencing to the investigation on oral microbiology indicates that communities of diverse organisms may be more critical than individual species in the development of oral diseases such as dental caries and periodontal diseases.^25^ Due to the polymicrobial infection nature of oral diseases, investigators and dental clinicians have realized that a comprehensive understanding of normal molecular baseline of the oral microbiome and the key factors that drive the compositional and functional shift of the microbial community are the prerequisites for accurate delineation of its pathogenic role in the onset and progression of oral diseases.^26^, ^27^

Since more than 50% of the oral microorganisms are unable to be cultivated,^28^ oral microbiome research, just like the microbiome research on other human body sites such as gut and skin, has been impeded by the intrinsic limitations of the conventional culture‐dependent methods for a long time. Accredited to the advancement in molecular biology, culture‐independent methods, such as fluorescence in situ hybridization,^29^ checkerboard DNA‐DNA hybridization method,^30^ 16S rRNA clone library analysis,^31^ terminal restriction fragment length polymorphism,^32^ denaturing gradient gel electrophoresis,^33^ and Human Oral Microbe Identification Microarray,^28^ have been employed to evaluate microbial diversity in various oral sites, substantially expanding the list of candidate pathogens associated with oral diseases. More importantly, the development of high‐throughput DNA sequencing technologies, such as 454 pyrosequencing (Roche Applied Science, Basel, Switzerland), Illumina MiSEquation (Illumina, San Diego, CA), SOLiD (Applied Biosystems, Foster City, CA), and Pacbio SMRT (PACIFIC BIOSCIENCES, Menlo Park, CA), has dramatically increased the resolution at which microbial communities can be analyzed.^34^

Of note, the launch of the Human Microbiome Project (HMP) (2007 to present) by the National Institute of Health of the United States, as well as other microbiome initiatives such as Metagenomics of the Human Intestinal Tract (2008‐2012) by the European Commission and International Human Microbiome Consortium (2017 to present), greatly promoted microbiome‐related research.^35^ However, current oral microbiome studies still hugely lag behind investigations on the gut microbiome. This phenomenon may partially be attributed to the shortage of funding in the oral research field. According to a report by the National Science and Technology Council Committee of the US, financial support for oral microbiome research over the fiscal years 2012‐2014 only accounted for less than 5% of total financial support on human microbiome‐related research.^36^ Another reason may be the lack of identification of leading researchers in this field. Rob Knight of University of California San Diego has been well recognized as one of the most active and productive investigators in gut microbiome research, and publications from leading groups like his may not only advance the science itself but also point out pressing needs and future direction of this field.

To comprehensively understand the current status, we adopted bibliometric analysis to qualitatively and quantitatively evaluate global oral microbiome‐related literature. We aimed to estimate the global scientific outputs of oral microbiome research to identify hotspots and trends, as well as the most active and productive investigators whose publications have imposed impact in this field and may influence the future direction of oral microbiome research.

## 2 METHODS AND MATERIALS

### 2.1 Data collection and processing

**FIGURE 1:**
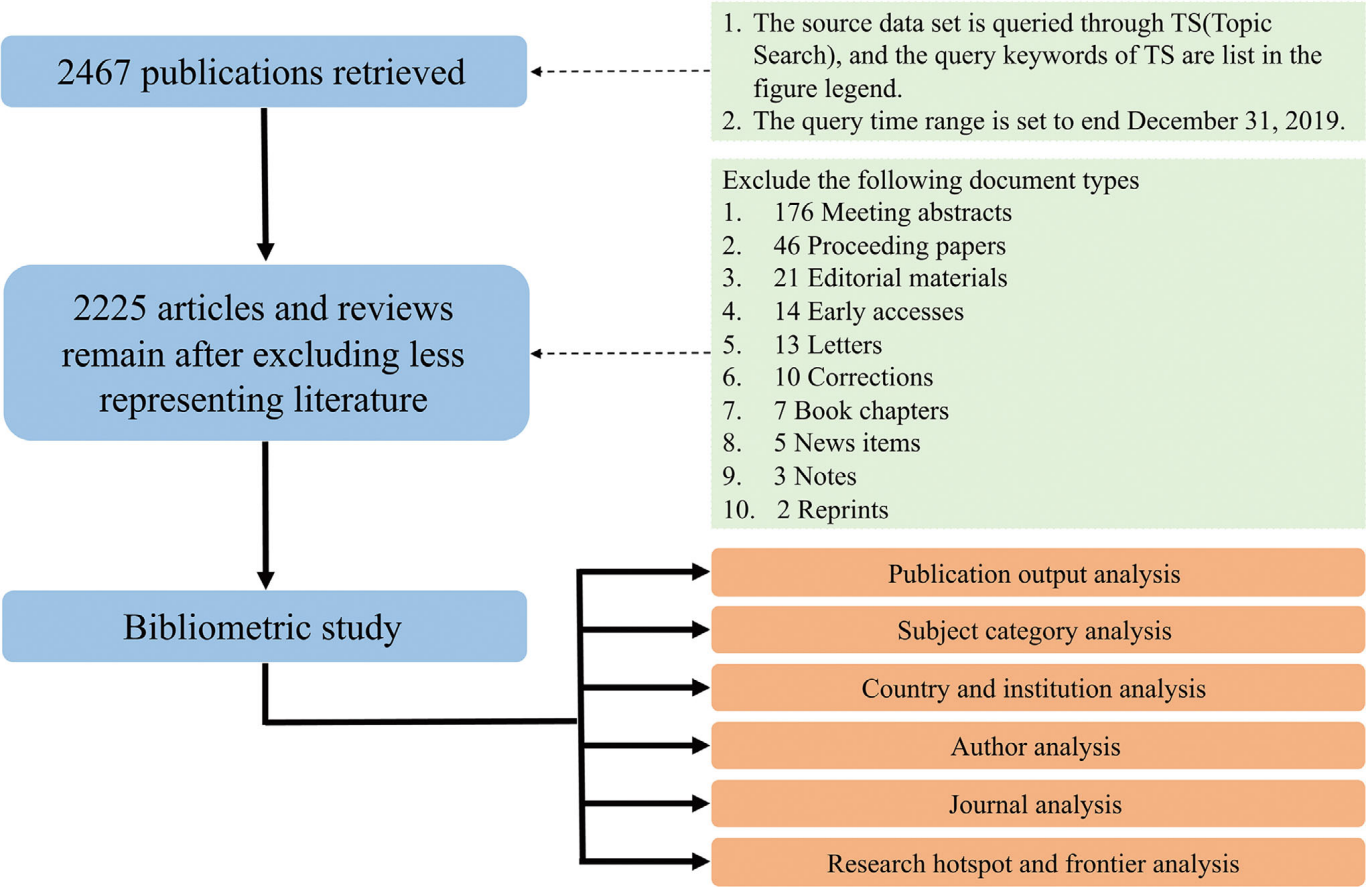
**The overall schematic diagram of this study**. Topic search (TS): “oral microbiome” or “oral microbiota” or “oral microflora” or “oral microbial community” or “oral microbial communities” or “dental microbiome” or “dental microbiota” or “dental microflora” or “dental microbial community” or “dental microbial communities” or “salivary microbiome” or “salivary microbiota” or “salivary microflora” or “salivary microbial community” or “salivary microbial communities” or “mouth microbiome” or “mouth microbiota” or “mouth microflora” or “mouth microbial community” or “mouth microbial communities”

According to our overall workflow (Figure 1), data were obtained from the most frequently used source for scientific literature Thomson Reuters' Web of Science Core Collection (WoSCC) which includes Science Citation Index (CI) Expanded, Emerging Sources CI (ESCI), Conference Proceedings CI‐Science (CPCI‐S), and Conference Proceedings CI‐Social Science & Humanities (CPCI‐SSH).

Sophisticated search terms were formed to generate the most accurate result dataset for subsequent analysis. All electronic searches were performed on the same day, January 20th, 2020. The whole records include titles, abstracts, author information, institutions, keywords, citations, references, et al. Our study systematically analyzed all the publications between January 1st, 1959 and December 31st, 2019, for depicting a landscape of evolution of oral microbiome research. All data were retrieved by two authors independently and stored in plain text format. The data were imported to CiteSpace and Microsoft Excel 2016 for subsequent analysis. All data were downloaded from the public database, and all data did not involve medical ethics issues.

### 2.2 Bibliometric analysis methods

In this study, we use bibliometrics methods to discover the history and development trends of oral microbiome‐related research. However, information on traditional bibliometrics research is relatively discrete. Therefore, in order to synthesize multidimensional information, on this basis, we have introduced the CiteSpace software for more comprehensive and visual analysis. We also used Python to generate the image to represent intricate middle layers information, including a collaborative connection network and cluster information. Based on the value of parameters like burst, centrality, and sigma, CiteSpace is a Java‐based scientific software package used for analyzing and visualizing co‐citation networks developed by Dr. Chaomei Chen.^37^ Burst measures a sudden change of items or citations, centrality quantifies the importance of the node's position in the network, and sigma is a combination of burst and centrality.^37^ This instrument allows researchers to observe and understand information efficiently to identify a model and the regularities of citations behind a mass date. CiteSpace V (64 bits) provides various functions for facilitating the understanding and interpretation of network patterns, including identifying the major topic areas, finding hotspots, and automatically labeling clusters with terms from selected literature.

In 1973, Marshakova and Small came up with the theory of document co‐citation, that is, when paper A and paper B are co‐cited by paper C at the same time, the relationship between paper A and paper B is a co‐citation relationship.^38^, ^39^

## 3 RESULTS

### 3.1 Characteristic of publication outputs

From 1959 to 2019, 2467 publications were obtained directly from WoSCC. In order to get a more accurate result in later analysis, fewer representing publications were excluded leaving only articles and reviews. The final dataset for later analysis included 2225 articles and reviews with a total of 58 912 references (Figure 1). Approximately 98.79% of the publications were written in English, indicating that English is the primary language used for communication among scholars, whereas the remaining 1.21% were written in other languages, Such as Spanish, French, German, etc.

**FIGURE 2:**
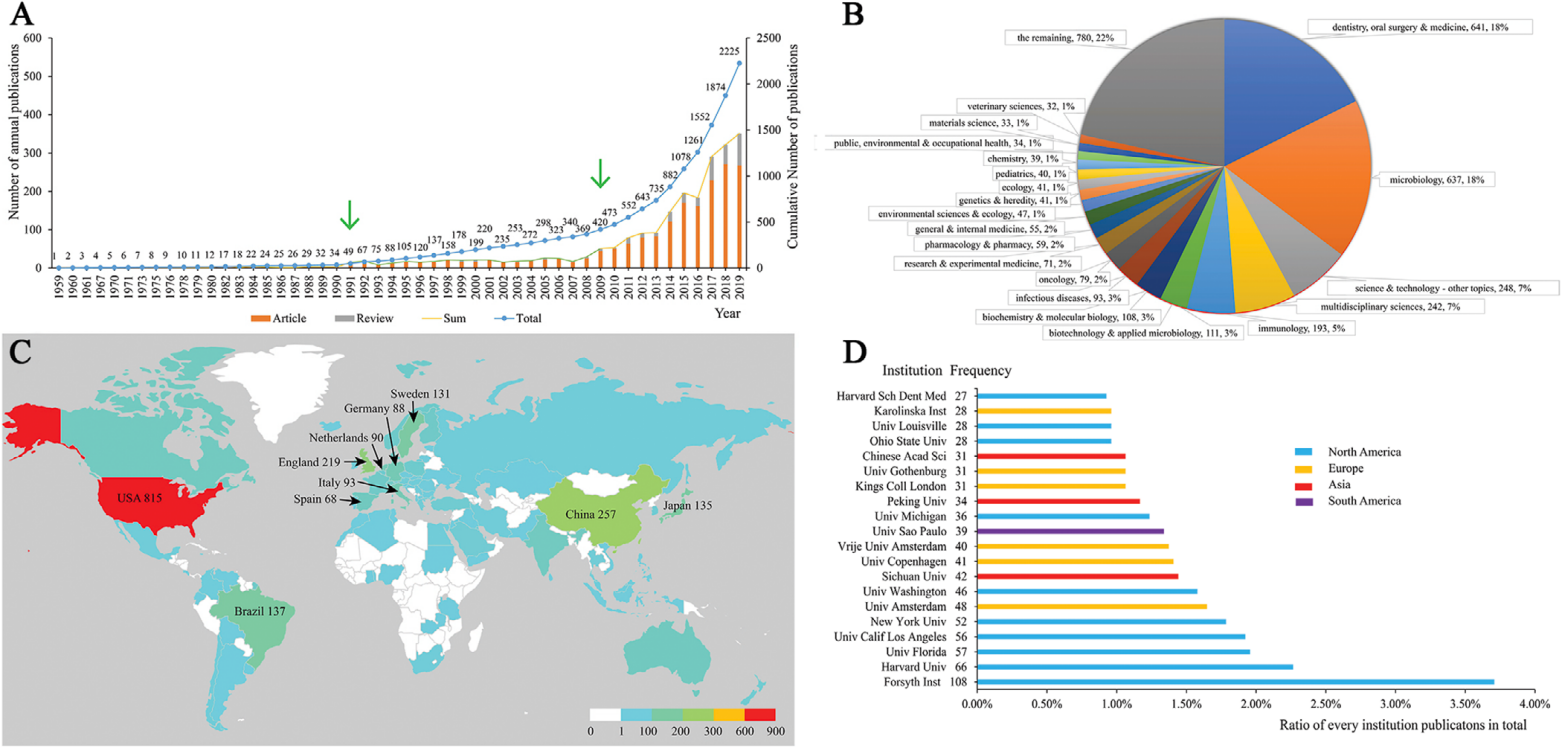
**Analysis of time, subject categories, countries, and institutions. A, Trends in the number of scholarly original articles and reviews** related to oral microbiome from 1959 to 2019. The green arrows separate three different stages according to the volume of publications. **B, Pie chart of subject categories** related to oral microbiome literatures from 1959 to 2019. **C, Geographic distribution of countries** on oral microbiome publications between 1959 and 2019. The colors of countries are corresponding to the frequencies according to the legend at the right bottom, and the top 10 countries and their frequencies are marked. **D, Top 20 institutions** related to oral microbiome literatures from 1959 to 2019. The frequencies are listed after the name of institutions, and different colors are corresponding to different continents

The distribution of annual publications is presented in Figure 2A by years. The overall trend of publication keeps increasing over time, and three distinct stages were noted according to the acceleration rate of the volume growth.

From 1959 to 1990, it was the embryonic stage of oral microbiome research. During this time stage, no annual publication volume exceeded 10 articles, the research fields were also very scattered, and no apparent research trend could be identified.

From 1991 to 2008, it was the development period of oral microbiome research. No significant number of articles were published annually, indicating there had been no major breakthrough during this period.

The period of 2009‐2019 was the outbreak of oral microbiome research. A large number of scientific literatures were published with the successful implementation of the HMP. A lot of unique research groups and research directions were formed during this stage. In 2018, more than 300 articles were published for the first time, which aroused much attention in academic circles.

### 3.2 Subject categories co‐occurrence analysis

The disciplines involved in the oral microbiome related to a specific knowledge domain can be detected based on the co‐occurrence analysis of the subject category. In our study, the 50 most reoccurring subject categories per year were selected for an analysis of category characteristics. CiteSpace was adopted to extract the subject category information from Supplement field tag of the WoS database for the subsequent analysis. Figure 2B shows the pie chart of all the subject categories, especially labeled the top 20. According to the values of parameters in Table S1, Figure S1 displays the co‐occurrence network from 1959 to 2019, where the node represents a subject category while an edge connecting two nodes demonstrates the co‐occurrence of the two subject categories. We can observe that dentistry, oral surgery and medicine (641) microbiology (637), and science and technology – other topics (248) are the top three popular categories of research, followed by multidisciplinary sciences (242) and immunology (193). The oral microbiome is a multifaceted and multidisciplinary field that covers a wide range of interests, from dentistry to microbiology, science, technology, etc.

Furthermore, some nodes indicate the high centrality values of the corresponding categories are represented by the purple rings.^40^ The top 50 productive subjects are shown in detail in Table S1. Of the top 50 subject categories, chemistry and biochemistry and molecular biology have the highest centrality and play an essential role in the oral microbiome.

### 3.3 Country cooperation analysis

The research groups in 98 countries published 2225 articles on oral microbiome publications (Figure 2C). The top 10 countries, including two American countries, two Asian countries, and six European countries, account for over 90% of the total number of literature (Table S2). On top of the list, the USA has published more than one‐third of the literature (36.6%), far ahead of other countries, followed by China (11.6%) and the United Kingdom (9.8%). The United States is also a center of cooperation worldwide, and cooperation has proliferated in recent years (Figure S2). Notably, P.R. China ranked in the top two in the list indicating it is fast progressing in oral microbiome science in recent years.

### 3.4 Institution cooperation analysis

Eight hundred and fourteen research institutes published these 2225 articles, indicating a broad interest in the oral microbiome. As can be seen from Figure 2D and the ranking list (Table S3), the top twenty organizations have published a total of 869 articles, accounting for nearly a third of the total, of which half are from North America, six are from Europe, and the remaining three in Asia and one in South America. Forsyth Institue and Harvard University have published the most articles, considering that Forsyth Institue is the affiliate of Harvard Medical School, which further demonstrates Harvard's outstanding contributions in this field. From Figure S3, we can find that the density of the overall network is low, indicating that there is still a need for enhanced cooperation between agencies.

### 3.5 Author analysis

**FIGURE 3:**
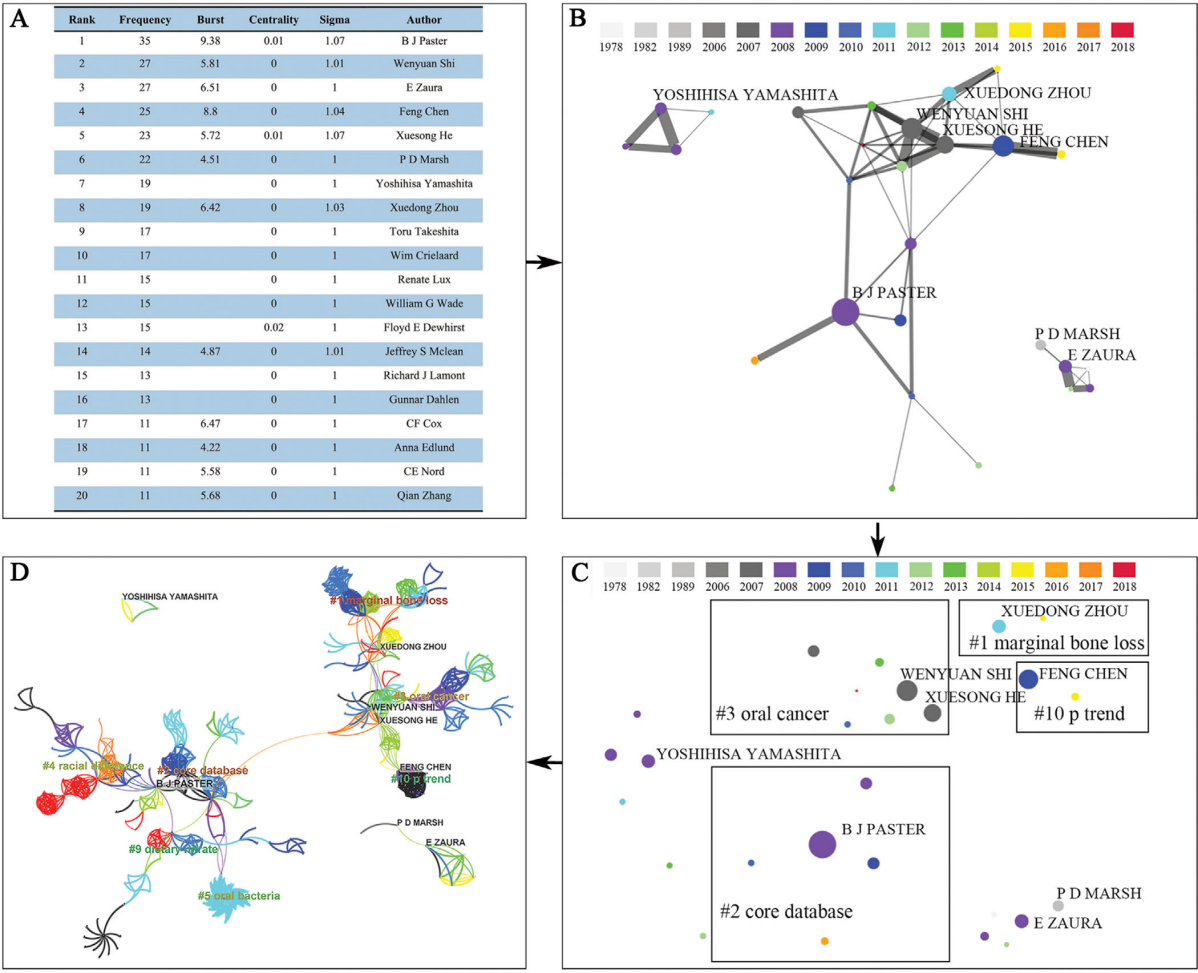
**Analysis of authors. A, Top 20 authors** on the oral microbiome literatures between 1959 and 2019. **B, Layer of collaborative connection information of authors** who published articles and reviews related to the oral microbiome from 1959 to 2019, and the image was generated by Python. Circular nodes represent authors, and the sizes of them are in proportion to the frequencies, and the colors of them are corresponding to the years in which they first published literature on the oral microbiome. The widths of lines are proportional to the number of publications that two authors both participated in. **C, Layer of cluster information of authors** who published articles and reviews related to the oral microbiome from 1959 to 2019, and the picture was produced by Python. The sequence number of main sub‐networks and cluster top terms are marked. **D, A visual clustering network of authors** published literature on oral microbiome between 1959 and 2019. Circular nodes represent authors, and the sizes of them are in proportion to the frequencies. The colors of links are corresponding to the year. The purple rims of circles represent the high centralities, and the red circles mean the high strength of bursts. The sequence number of sub‐networks and cluster top terms are marked in the central area. The larger the number of authors includes in the cluster, the more the label color becomes warm

More than 3000 authors published articles related to the oral microbiome, and Table S4 shows the top 50 authors based on the frequency, and Paster BJ is ranked first. Among the top ten, there are four Chinese scientists. As shown in Figure 3A, we display the top twenty authors and their related parameters; based on this data, we use Python to produce Figure 3B, 3C to represent collaborative network and research topic clusters of authors with high frequencies. We use the abstract terms to perform LLR clustering and get two vast clustering networks (Figure 3D). The CiteSpace configuration: link retaining factor (LRF = 2), look back years (LBY = 8), e for top N (e = 2, N = 50), time span (1959‐2019), and years per slice (1). The clustering network on the upper right is centered on four Chinese scientists Wenyuan Shi, Feng Chen, Xuesong He, and Xuedong Zhou whose main research direction is marginal bone loss and oral cancer; the clustering network in the lower‐left corner is centered on BJ Paster whose main research direction is the core database. The connection between the two main clustering networks is orange, indicates that the two major clusters began to cross in recent years. The larger the number of authors includes in the cluster, the more the label color becomes warm. Additionally, we noted that PD Marsh, E Zaura, and Yoshihisa Yamashita are ranked in the top ten, but they form a tiny category in individual units.

### 3.6 Co‐cited author analysis

**FIGURE 4:**
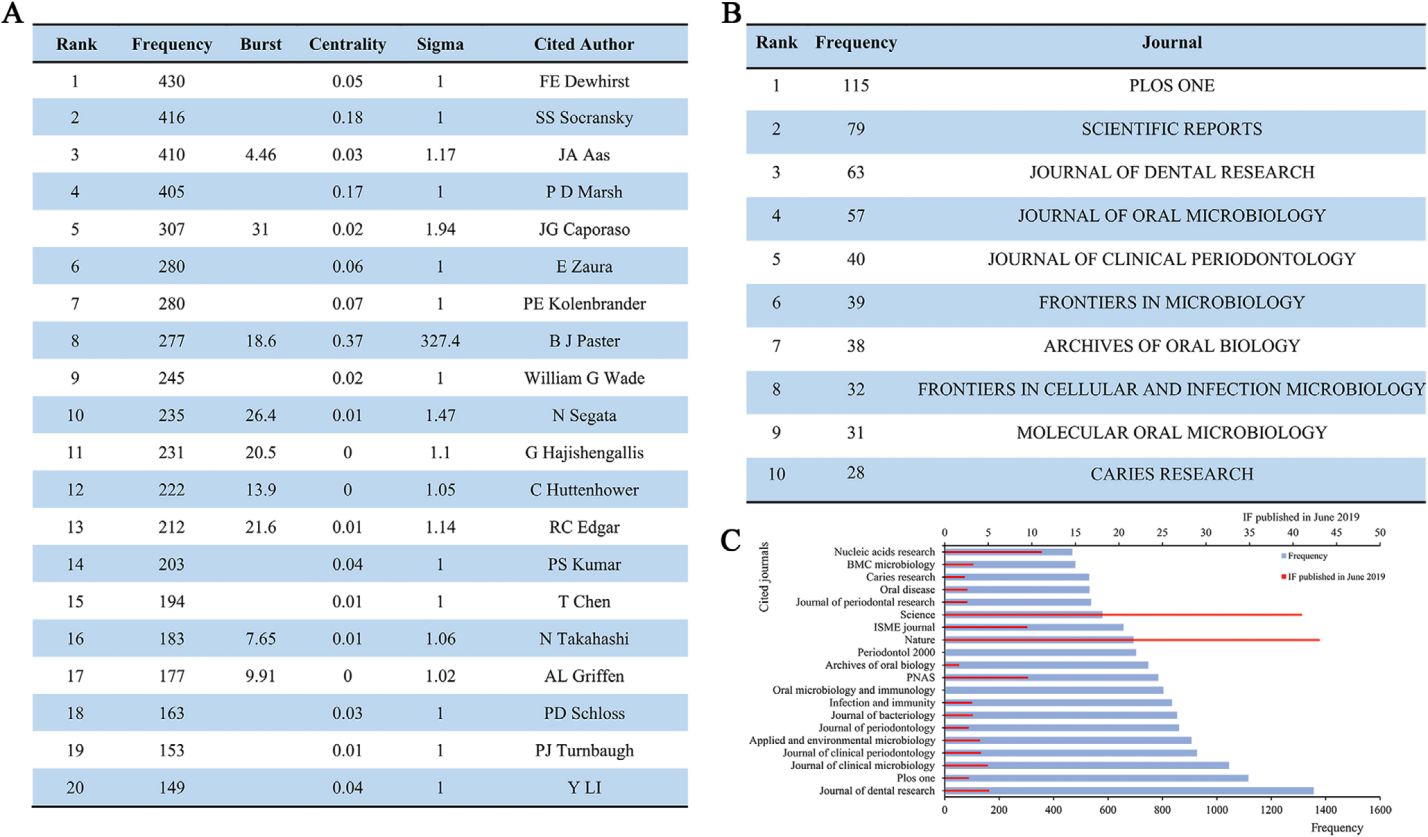
**Analysis of co‐cited authors, journals, and cited journals. A, Top 20 co‐cited authors** on the oral microbiome literatures between 1959 and 2019. According to the parameters above, 704 cited‐authors structured the network, and the top 20 are listed. Burst measures a sudden change of items or citations, centrality quantifies the importance of the node's position in the network, and sigma is a combination of burst and centrality. **B, Top 10 journals** in terms of frequency. **C, Top 20 cited journals** in the oral microbiome publications between 1959 and 2019. Impact factor list is according to the one published in June 2019

Nowadays, citation networks are widely used in information science visual analysis. Here, we used Citespace V to analyze the author's citations in the oral microbiome study and constructed a network to estimate the scientific relevance of the publication. As shown in Figure 4A and Figure S4, the largest nodes including FE Dewhirst (430 citations), SS Socransky (416 citations), JA Aas (410 citations), PD Marsh (405 citations), and JG Caporaso (307 citations), which indicate their high impact in the oral microbiome research. PD Marsh, BJ Paster, and E Zaura are both high ranking authors and highly cited authors, indicating that the three people not only published a large number of articles but also had high quality. It is suggested that we should not only focus on quantity in future research but, more importantly, improve research quality.

### 3.7 Journal analysis

In order to understand the status of published journals related to oral microbiome research, we analyzed the frequency of publication of these articles in various journals through WoSCC. The top ten journals are listed in Figure 4B. The top three are Plos One (IF = 2.776), Scientific Reports (IF = 4.011), and Journal of Dental Research (IF = 5.125). Besides, to demonstrate the impact of journals in this field better, we used CiteSpace to analyze the cited frequency of articles in this field in the journal and generated a high‐definition image by Pathfinder network scaling, which enhances the clarity of the fusion network. Figure S5 shows the journal network with high citations. Figure 4C exhibits the top twenty cited journals with the highest frequency of citations and their impact factors published in June 2019, also Table S5 lists the top fifty with more details. The Journal of Dental Research topped the list with a frequency of 1356, which is positively correlated with its high impact factor. In summary, it has been shown that the Journal of Dental Research has had a profound impact on oral microbiology research, both in terms of publication and citation.

### 3.8 Research hotspot

Each research article cites many references. The analysis of references is one of the most significant indicators of bibliometrics. The hypothesis is that if one reference is usually cited along with another, it is distinct that they are associated in some ways.^39^, ^41^, ^42^ In CiteSpace, the nodes labeled with years and corresponding authors represented different references, and the co‐citation network was divided into various clusters (Figure S6). Each cluster represents a thematic concentration or an evident specialty and is labeled by noun phrases from titles of citing articles.^37^, ^41^

To some extent, using terms from citing articles is due to the limitation of source data, and titles of cited references may not always be available from the WoSCC. Clusters with few members tend to be less representative than the larger ones because small clusters are probably to be formed by the citing behavior of a small number of articles. For example, #0 dysbiosis, as the largest cluster, contains not only six of the top 10 cited documents but also a large number of cited documents with new co‐citation time (hot color links). Moreover, we can find that evolution is generally from left to right in the network, from related‐technology to new hotspots like biofilms and periodontal disease (Figure S6). Notably, we found a new cluster #6 pancreatic cancer with red lines. Pancreatic cancer is a common malignancy of the digestive tract, and the oral is the initial part of the digestive tract. The associations between oral diseases and increased risk of pancreatic cancer have been reported in several studies.^43^, ^44^

On the other hand, keywords offer a reasonable presentation of research hotspots, keeping an eye by researchers on many relevant questions and concepts. According to frequencies, we identified the top 20 keywords (Table S6), and during this process, keywords with the same meaning were combined, and the following is a conventional analysis. From the table, we can observe that periodontal disease, oral microbes, and dental plaque are at the forefront, which is consistent with the results of the cited literature clustering map. Therefore, we believe that the above several directions are research hotspots.

### 3.9 Research frontier

**FIGURE 5:**
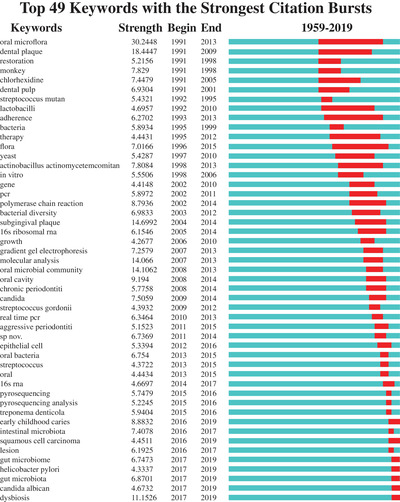
**The keywords with the strongest citation bursts of articles on oral microbiome publications from 1959 to 2019**. The time interval is depicted as a blue line and the period that represents a burst keyword as a red line, indicating the beginning and the ending of the time interval of each burst

Meanwhile, burst words of keywords behave on new research frontiers (sudden changes and emerging trends that occur in time).^45^, ^46^ Moreover, we used CiteSpace to construct a knowledge map of keyword co‐occurrence to find a burst of the keywords. The keywords with the strongest citation bursts recently are listed in Figure 5 from which we can observe the keyword evolution over time and the most current burst keywords, including early childhood caries, squamous cell carcinoma, gut microbiome, *Helicobacter pylori*, *Candida albicans*, and dysbiosis.

## 4 DISCUSSION

This study investigates the global scientific outputs of the oral microbiome until December 31st, 2019. We analyzed data obtained from the WoSCC on multiple perspectives: publication outputs, countries, institutions, authors, co‐cited journals, and keywords. We discovered that researchers paid more attention to this field in recent years, according to the annual publications. Furthermore, the USA is the most active contributor with the most publications and cooperation among all the countries. As far as authors are concerned, BJ Paster appeared in the top, reflecting his contributions to this area, while considered cited‐authors, FE Dewhirst, SS Socransky, and JA Aas ranked top. Among the top ten scientists, there are four Chinese scientists, and they have formed a massive network of active cooperation, indicating that Chinese scientists are very active and contributing in this field recently. The Journal of Dental Research has had a profound impact on oral microbiology research, both in terms of publication and citation.

We also found that current research hotspots include: periodontal disease, oral microbes, and dental plaque. In traditional research, most studies were standalone. With the passage of time, the advancement of technology, the new understanding of the disease mechanism, the pursuit of precision medicine, different research fields gradually cross. In recent years, researchers have found that the oral microbiome actually plays an essential biological role in the way of interacting with human and oral health to form an interactive network and regularly exchanges signals and substances,^47^, ^48^ such as oral microbes and human secretions.^49^ For example, the oral microbiome is related to the saliva and constantly interacts with saliva,^50^ which not only causes the formation of plaque but is also closely related to other diseases.^11^ It also confirms the hotspots results of our research.

A series of burst keywords discovered by our research, including early childhood caries, squamous cell carcinoma, gut microbiome, *Helicobacter pylori*, *Candida albicans*, and dysbiosis, have a high possibility of becoming the next stage of research hotspots: (1) For younger children, caries is identified as a burst keyword, because children and adults are at different stages of life, and the difference in the endocrine system, diet, lifestyle, and immunity between them will have a huge impact on oral health.^51^, ^52^ Subspecialties formation is crucial for precise treatment and health maintenance.^53^ (2) Scholars now believe that cancer is a complex disease caused by genetic factors and environmental factors.^54^, ^55^ Microorganisms, because of their large number, wide variety, wide distribution, long‐term coexistence with humans, are important environmental factors.^56^, ^57^ Interactions might cause common diseases, such as squamous cell carcinoma^58^ and pancreatic cancer.^59^ (3) Oral is the entrance to the digestive tract, oral microbes are also the primary source of human digestive flora,^60^, ^61^ as the Chinese proverb says: “the disease is from the mouth,” so for the oral microbiome research, we cannot only focus on the oral cavity but also should pay attention to the entire digestive tract, including microbes like *Helicobacter pylori*, found on the human gastric mucosa, because, in any position of the digestive tract, the oral microbiome may play an essential biological role,^12^ affecting the function of the whole digestion,^62^ thus affecting human health.^11^ (4) Recently the study of the oral microbiota mainly focused on bacteria due to relatively high abundance and easy detection. However, with the researches on fungal species that inhabit the human mouth increasing, we find *Candida albicans*, as an overlooked contributor, play a significant role in fungal‐bacterial interactions.^10^, ^63^, ^64^ (5) The human mouth provides bacteria, viruses, archaea, and fungi that reside in complex and polymicrobial communities. This oral microbiome can remain in mutualistic balance with the host or can cause dysbiosis resulting in an increased risk of diseases, such as dental caries and periodontitis.^64^, ^65^, ^66^

Nevertheless, compared with the traditional articles from domain experts, the analyses in this paper have certain limitations, for example, the first name and last name of authors cannot be distinguished clearly in the map generated by CiteSpace.

Therefore, through bibliometric research, we can locate hotspots and frontiers in related fields accurately, and it is easier to discover interdisciplinary development. The knowledge map formed by big data can provide much valuable information for practitioners in the field, including related scientists, students, journals, editors, etc. which enables us to precisely using the oral microbiology group to remove its adverse effects, enhance its beneficial effects, and ultimately improve human oral health.

## CONFLICT OF INTEREST

The authors declare that there is no conflict of interest that could be perceived as prejudicing the impartiality of the research reported.

## AUTHOR CONTRIBUTIONS

Ga Liao and Xuedong Zhou initiated the project and designed the experiments. Jinyun Wu and Li Tang conducted the bibliometric study. Yuqing Li, Xian Peng, Xin Xu, and Dongmei Deng interpreted the data. Ga Liao and Jinyun Wu prepared the manuscript. All authors participated in revising the manuscript and agreed to the final version.

## Supporting information

SUPPORTING INFORMATION

## Data Availability

Research data are available from the corresponding author upon reasonable request.
