# Supplementary material for: Visualized analysis of trends and hotspots in global oral microbiome research: A bibliometric study
Source: MedComm (2020). 2020 Dec 10;1(3):351–61. doi: 10.1002/mco2.47 (PMC8491219; doi:10.1002/mco2.47)
Supplement: Supplementary file 1 — SUPPORTING INFORMATION [file MCO2-1-351-s001.docx]

**Title: Visualized analysis of trends and hotspots in global oral microbiome research: A bibliometric study**

**Supplementary Tables:**

**Table S1: Top 50 research areas related to oral microbiome publications between 1959 and 2019.** The CiteSpace parameters were as follows: link retaining factor (LRF = 2), look back years (LBY = 8), e for top N (e = 1, N = 50), time span (1959–2019), and years per slice (1). Among 130 research areas, the top 50 were list below. Burst measures a sudden change of items or citations, centrality quantifies the importance of the node’s position in the network and sigma is a combination of burst and centrality.

| **Rank** | | **Frequency** | **Burst** | **Centrality** | **Sigma** | **Research area** |
| --- | --- | --- | --- | --- | --- | --- |
| 1 | 641 | 51.11 | 0.17 | 3291.59 | DENTISTRY, ORAL SURGERY & MEDICINE |  |
| 2 | 637 |  | 0.09 | 1 | MICROBIOLOGY |  |
| 3 | 248 |  | 0.03 | 1 | SCIENCE & TECHNOLOGY - OTHER TOPICS |  |
| 4 | 242 |  | 0 | 1 | MULTIDISCIPLINARY SCIENCES |  |
| 5 | 193 | 9.27 | 0.15 | 3.69 | IMMUNOLOGY |  |
| 6 | 111 |  | 0.1 | 1 | BIOTECHNOLOGY & APPLIED MICROBIOLOGY |  |
| 7 | 108 |  | 0.25 | 1 | BIOCHEMISTRY & MOLECULAR BIOLOGY |  |
| 8 | 93 | 5.62 | 0.04 | 1.22 | INFECTIOUS DISEASES |  |
| 9 | 79 |  | 0.2 | 1 | ONCOLOGY |  |
| 10 | 71 |  | 0.09 | 1 | RESEARCH & EXPERIMENTAL MEDICINE |  |
| 11 | 59 |  | 0.02 | 1 | PHARMACOLOGY & PHARMACY |  |
| 12 | 55 |  | 0.05 | 1 | GENERAL & INTERNAL MEDICINE |  |
| 13 | 47 |  | 0.01 | 1 | ENVIRONMENTAL SCIENCES & ECOLOGY |  |
| 14 | 41 |  | 0.01 | 1 | GENETICS & HEREDITY |  |
| 15 | 41 |  | 0 | 1 | ECOLOGY |  |
| 16 | 40 |  | 0.06 | 1 | PEDIATRICS |  |
| 17 | 39 |  | 0.27 | 1 | CHEMISTRY |  |
| 18 | 34 |  | 0.05 | 1 | PUBLIC, ENVIRONMENTAL & OCCUPATIONAL HEALTH |  |
| 19 | 33 |  | 0.11 | 1 | MATERIALS SCIENCE |  |
| 20 | 32 |  | 0.03 | 1 | VETERINARY SCIENCES |  |
| 21 | 32 |  | 0.02 | 1 | GASTROENTEROLOGY & HEPATOLOGY |  |
| 22 | 30 |  | 0.03 | 1 | NUTRITION & DIETETICS |  |
| 23 | 29 |  | 0.15 | 1 | CELL BIOLOGY |  |
| 24 | 29 |  | 0.05 | 1 | BIOCHEMICAL RESEARCH METHODS |  |
| 25 | 27 |  | 0.03 | 1 | FOOD SCIENCE & TECHNOLOGY |  |
| 26 | 25 |  | 0.01 | 1 | ENDOCRINOLOGY & METABOLISM |  |
| 27 | 22 |  | 0.01 | 1 | PATHOLOGY |  |
| 28 | 21 |  | 0.02 | 1 | NEUROSCIENCES & NEUROLOGY |  |
| 29 | 20 |  | 0.03 | 1 | MATERISLA SCIENCE, BIOMATERIALS |  |
| 30 | 19 |  | 0.08 | 1 | ENGINEERING |  |
| 31 | 18 |  | 0 | 1 | VIROLOGY |  |
| 32 | 18 |  | 0 | 1 | PARASITOLOGY |  |
| 33 | 17 |  | 0.05 | 1 | ENGINEERING, BIOMEDICAL |  |
| 34 | 16 |  | 0.01 | 1 | NEUROSCIENCES |  |
| 35 | 15 |  | 0 | 1 | OBSTETRICS & GYNECOLOGY |  |
| 36 | 15 |  | 0.05 | 1 | BIOLOGY |  |
| 37 | 15 |  | 0.01 | 1 | CARDIOVASCULAR SYSTEM & CARDIOLOGY |  |
| 38 | 15 |  | 0 | 1 | MARINE & FRESHWATER BIOLOGY |  |
| 39 | 15 |  | 0.05 | 1 | LIFE SCIENCES & BIOMEDICINE - OTHER TOPICS |  |
| 40 | 13 |  | 0.01 | 1 | CHEMISTRY, MULTIDISCIPLINARY |  |
| 41 | 12 |  | 0 | 1 | MYCOLOGY |  |
| 42 | 12 |  | 0.01 | 1 | CHEMISTRY, APPLIED |  |
| 43 | 11 |  | 0 | 1 | HEALTH CARE SCIENCES & SERVICES |  |
| 44 | 11 |  | 0.03 | 1 | GERIATRICS & GERONTOLOGY |  |
| 45 | 11 |  | 0 | 1 | MATERIALS SCIENCE, MULTIDISCIPLINARY |  |
| 46 | 11 |  | 0 | 1 | CHEMISTRY, MEDICINAL |  |
| 47 | 11 |  | 0.1 | 1 | RESPIRATORY SYSTEM |  |
| 48 | 11 |  | 0 | 1 | HEALTH CARE SCIENCES & SERVICES |  |
| 49 | 10 |  | 0.05 | 1 | SURGERY |  |
| 50 | 10 |  | 0 | 1 | CLINICAL NEUROLOGY |  |

**Table S2: The top 50 active countries on the oral microbiome publications from 1959 to 2019.** The CiteSpace parameters were as follows: link retaining factor (LRF = 2), look back years (LBY = 8), e for top N (e = 1, N = 50), time span (1959–2019), and years per slice (1). The research groups in 98 countries published articles and the top 50 were list below. Burst measures a sudden change of items or citations, centrality quantifies the importance of the node’s position in the network and sigma is a combination of burst and centrality.

| **Rank** | **Frequency** | **Burst** | **Centrality** | **Sigma** | **Country** |
| --- | --- | --- | --- | --- | --- |
| 1 | 815 |  | 0.39 | 1 | USA |
| 2 | 257 |  | 0.08 | 1 | PEOPLES R CHINA |
| 3 | 219 | 12.6 | 0.11 | 3.93 | ENGLAND |
| 4 | 137 | 9.39 | 0.01 | 1.06 | BRAZIL |
| 5 | 135 |  | 0.06 | 1 | JAPAN |
| 6 | 131 | 16.4 | 0.14 | 7.99 | SWEDEN |
| 7 | 93 |  | 0.09 | 1 | ITALY |
| 8 | 90 |  | 0.07 | 1 | NETHERLANDS |
| 9 | 88 |  | 0.12 | 1 | GERMANY |
| 10 | 68 |  | 0.15 | 1 | SPAIN |
| 11 | 68 | 5.63 | 0.09 | 1.61 | AUSTRALIA |
| 12 | 67 |  | 0.1 | 1 | CANADA |
| 13 | 66 | 5.62 | 0.01 | 1.03 | INDIA |
| 14 | 55 |  | 0.03 | 1 | DENMARK |
| 15 | 53 |  | 0.17 | 1 | SWITZERLAND |
| 16 | 45 |  | 0.11 | 1 | FRANCE |
| 17 | 45 | 4.78 | 0.09 | 1.52 | FINLAND |
| 18 | 43 | 5.97 | 0.03 | 1.22 | NORWAY |
| 19 | 37 |  | 0.01 | 1 | POLAND |
| 20 | 32 |  | 0.05 | 1 | BELGIUM |
| 21 | 29 |  | 0.01 | 1 | NEW ZEALAND |
| 22 | 28 |  | 0 | 1 | TURKEY |
| 23 | 26 |  | 0 | 1 | SOUTH KOREA |
| 24 | 23 |  | 0.01 | 1 | ISRAEL |
| 25 | 23 |  | 0.07 | 1 | SCOTLAND |
| 26 | 20 |  | 0 | 1 | SAUDI ARABIA |
| 27 | 19 |  | 0 | 1 | WALES |
| 28 | 18 |  | 0.01 | 1 | THAILAND |
| 29 | 16 |  | 0.03 | 1 | SINGAPORE |
| 30 | 15 |  | 0.01 | 1 | GREECE |
| 31 | 14 |  | 0.01 | 1 | AUSTRIA |
| 32 | 14 |  | 0 | 1 | TAIWAN |
| 33 | 12 |  | 0.01 | 1 | PORTUGAL |
| 34 | 12 |  | 0 | 1 | IRAN |
| 35 | 12 |  | 0 | 1 | IRELAND |
| 36 | 11 |  | 0 | 1 | MEXICO |
| 37 | 10 |  | 0.03 | 1 | CHILE |
| 38 | 9 |  | 0 | 1 | HUNGARY |
| 39 | 9 |  | 0 | 1 | EGYPT |
| 40 | 8 |  | 0.03 | 1 | SOUTH AFRICA |
| 41 | 8 |  | 0 | 1 | RUSSIA |
| 42 | 7 |  | 0 | 1 | MALAYSIA |
| 43 | 7 |  | 0 | 1 | COLOMBIA |
| 44 | 6 |  | 0 | 1 | KUWAIT |
| 45 | 5 |  | 0 | 1 | ROMANIA |
| 46 | 5 |  | 0 | 1 | INDONESIA |
| 47 | 5 |  | 0 | 1 | CZECH REPUBLIC |
| 48 | 4 |  | 0 | 1 | SERBIA |
| 49 | 4 |  | 0 | 1 | BULGARIA |
| 50 | 4 |  | 0 | 1 | U ARAB EMIRATES |

**Table S3: The top 50 active institutions on oral microbiome literatures from 1959 to 2019.** The CiteSpace parameters were as follows: link retaining factor (LRF = 2), look back years (LBY = 8), e for top N (e = 1, N = 50), time span (1959–2019), and years per slice (1). Among the overall 814 institutions, the top 50 were list below. Burst measures a sudden change of items or citations, centrality quantifies the importance of the node’s position in the network and sigma is a combination of burst and centrality.

| **Rank** | **Frequency** | **Burst** | **Centrality** | **Sigma** | **Institution** |
| --- | --- | --- | --- | --- | --- |
| 1 | 108 | 5.3 | 0.12 | 1.81 | Forsyth Inst |
| 2 | 66 | 9.59 | 0.09 | 2.35 | Harvard Univ |
| 3 | 57 |  | 0.09 | 1 | Univ Florida |
| 4 | 56 |  | 0.04 | 1 | Univ Calif Los Angeles |
| 5 | 52 |  | 0.05 | 1 | NYU |
| 6 | 48 |  | 0.06 | 1 | Univ Amsterdam |
| 7 | 46 |  | 0.04 | 1 | Univ Washington |
| 8 | 42 | 5.68 | 0.02 | 1.13 | Sichuan Univ |
| 9 | 41 | 5.93 | 0.02 | 1.13 | Univ Copenhagen |
| 10 | 40 |  | 0.03 | 1 | Vrije Univ Amsterdam |
| 11 | 39 |  | 0.03 | 1 | Univ Sao Paulo |
| 12 | 36 |  | 0.02 | 1 | Univ Michigan |
| 13 | 34 |  | 0.01 | 1 | Peking Univ |
| 14 | 31 |  | 0.07 | 1 | Kings Coll London |
| 15 | 31 |  | 0.03 | 1 | Univ Gothenburg |
| 16 | 31 | 4.67 | 0.03 | 1.15 | Chinese Acad Sci |
| 17 | 28 |  | 0.02 | 1 | Ohio State Univ |
| 18 | 28 |  | 0.06 | 1 | Univ Louisville |
| 19 | 28 |  | 0.03 | 1 | Karolinska Inst |
| 20 | 27 | 8.97 | 0.01 | 1.05 | Harvard Sch Dent Med |
| 21 | 26 | 7.93 | 0.01 | 1.06 | Univ Penn |
| 22 | 25 |  | 0 | 1 | J Craig Venter Inst |
| 23 | 24 |  | 0.01 | 1 | SUNY Buffalo |
| 24 | 23 |  | 0.02 | 1 | Kyushu Univ |
| 25 | 23 | 5.77 | 0.01 | 1.05 | Univ Queensland |
| 26 | 22 |  | 0.03 | 1 | Univ Calif San Francisco |
| 27 | 21 |  | 0 | 1 | Shanghai Jiao Tong Univ |
| 28 | 19 |  | 0.04 | 1 | Univ Helsinki |
| 29 | 19 |  | 0.01 | 1 | Univ Hong Kong |
| 30 | 17 | 6.78 | 0.02 | 1.16 | Queen Mary Univ London |
| 31 | 17 |  | 0.01 | 1 | Univ Oslo |
| 32 | 17 | 6.42 | 0.05 | 1.33 | UCL |
| 33 | 16 | 6.5 | 0.02 | 1.13 | Univ Milan |
| 34 | 16 |  | 0.01 | 1 | Univ Minnesota |
| 35 | 14 |  | 0.01 | 1 | Univ Groningen |
| 36 | 14 |  | 0.01 | 1 | Univ Maryland |
| 37 | 14 |  | 0.01 | 1 | Univ Oklahoma |
| 38 | 14 |  | 0.01 | 1 | Zhejiang Univ |
| 39 | 14 |  | 0 | 1 | Virginia Commonwealth Univ |
| 40 | 13 |  | 0.01 | 1 | Univ Ghent |
| 41 | 13 |  | 0 | 1 | Umea Univ |
| 42 | 13 |  | 0.01 | 1 | Univ Otago |
| 43 | 13 |  | 0.01 | 1 | Univ Estadual Campinas |
| 44 | 13 | 4.7 | 0 | 1.01 | Univ N Carolina |
| 45 | 13 |  | 0 | 1 | Univ Bern |
| 46 | 12 |  | 0 | 1 | Univ Fed Minas Gerais |
| 47 | 12 |  | 0.01 | 1 | Univ Zurich |
| 48 | 12 | 6.21 | 0.01 | 1.07 | NCI |
| 49 | 11 | 4.71 | 0.01 | 1.03 | Univ Calif San Diego |
| 50 | 10 |  | 0.01 | 1 | Tohoku Univ |

**Table S4: The top 50 active authors related to oral microbiome publications from 1959 to 2019.** The CiteSpace parameters were as follows: link retaining factor (LRF = 2), look back years (LBY = 8), e for top N (e = 1, N = 50), time span (1959–2019), and years per slice (1). There are 3266 authors published articles and according to the frequency, the top 50 active authors were list below. Burst measures a sudden change of items or citations, centrality quantifies the importance of the node’s position in the network and sigma is a combination of burst and centrality.

| **Rank** | **Frequency** | **Burst** | **Centrality** | **Sigma** | **Author** |
| --- | --- | --- | --- | --- | --- |
| 1 | 35 | 9.38 | 0.01 | 1.07 | Paster BJ |
| 2 | 27 | 5.81 | 0 | 1.01 | Shi WY |
| 3 | 27 | 6.51 | 0 | 1 | Zaura E |
| 4 | 25 | 8.8 | 0 | 1.04 | Chen F |
| 5 | 23 | 5.72 | 0.01 | 1.07 | He XS |
| 6 | 22 | 4.51 | 0 | 1 | Marsh PD |
| 7 | 19 |  | 0 | 1 | Yamashita Y |
| 8 | 19 | 6.42 | 0 | 1.03 | Zhou XD |
| 9 | 17 |  | 0 | 1 | Takeshita T |
| 10 | 17 |  | 0 | 1 | Crielaard W |
| 11 | 15 |  | 0 | 1 | Lux R |
| 12 | 15 |  | 0 | 1 | Wade W |
| 13 | 15 |  | 0.02 | 1 | Dewhirst FE |
| 14 | 14 | 4.87 | 0 | 1.01 | Mclean JS |
| 15 | 13 |  | 0 | 1 | Lamont RJ |
| 16 | 13 |  | 0 | 1 | Dahlen G |
| 17 | 11 | 6.47 | 0 | 1 | Cox C |
| 18 | 11 | 4.22 | 0 | 1 | Edlund A |
| 19 | 11 | 5.58 | 0 | 1 | Nord C |
| 20 | 11 | 5.68 | 0 | 1 | Zhang Q |
| 21 | 10 | 4.86 | 0 | 1 | Belstrom D |
| 22 | 10 |  | 0 | 1 | Buijs MJ |
| 23 | 10 |  | 0 | 1 | Shibata Y |
| 24 | 9 |  | 0 | 1 | Kumar PS |
| 25 | 9 |  | 0 | 1 | Friaslopez J |
| 26 | 9 |  | 0 | 1 | Nelson KE |
| 27 | 9 |  | 0 | 1 | Xu X |
| 28 | 9 |  | 0 | 1 | Chen T |
| 29 | 8 |  | 0 | 1 | Chen H |
| 30 | 8 |  | 0 | 1 | Watt RM |
| 31 | 8 |  | 0 | 1 | Leys EJ |
| 32 | 8 | 4.86 | 0 | 1 | Wilson M |
| 33 | 8 |  | 0 | 1 | Olsen I |
| 34 | 8 |  | 0 | 1 | Brandt BW |
| 35 | 8 |  | 0 | 1 | Hayes RB |
| 36 | 8 |  | 0 | 1 | Ahn J |
| 37 | 7 |  | 0 | 1 | Potempa J |
| 38 | 7 |  | 0 | 1 | Huang Z |
| 39 | 7 |  | 0 | 1 | Guo L |
| 40 | 7 |  | 0 | 1 | Huang S |
| 41 | 7 | 4.42 | 0 | 1 | Suzuki S |
| 42 | 7 |  | 0 | 1 | Keijser B |
| 43 | 7 |  | 0 | 1 | Li Y |
| 44 | 7 |  | 0 | 1 | Beighton D |
| 45 | 7 |  | 0 | 1 | Pei Z |
| 46 | 7 | 4.26 | 0 | 1 | Dahlen G |
| 47 | 7 | 4.25 | 0 | 1 | Mullany P |
| 48 | 6 |  | 0 | 1 | Velsko IM |
| 49 | 6 |  | 0 | 1 | Mertas A |
| 50 | 6 |  | 0 | 1 | Pride DT |

**Table S5: The top 50 co-cited journals related to oral microbiome literatures from 1959 to 2019 and IF published in June 2019.** The CiteSpace configuration: link retaining factor (LRF = 2), look back years (LBY = 8), e for top N (e = 2, N = 50), time span (1959–2019), and years per slice (1). According to the parameters above, 329 cited-journals structured the network and the top 50 were list below. Burst measures a sudden change of items or citations, centrality quantifies the importance of the node’s position in the network and sigma is a combination of burst and centrality.

| **Rank** | **Frequency** | **Burst** | | **Centrality** | | **Sigma** | | **Cited Journal** | **IF 2019** | |
| --- | --- | --- | --- | --- | --- | --- | --- | --- | --- | --- |
| 1 | 1356 | 11.9 | 0.06 | | 2 | | JOURNAL OF DENTAL RESEARCH | | | 5.125 |
| 2 | 1116 |  | 0.06 | | 1 | | PLOS ONE | | | 2.776 |
| 3 | 1045 |  | 0.08 | | 1 | | JOURNAL OF CLINICAL MICROBIOLOGY | | | 4.959 |
| 4 | 927 |  | 0.07 | | 1 | | JOURNAL OF CLINICAL PERIODONTOLOGY | | | 4.164 |
| 5 | 907 |  | 0.03 | | 1 | | APPLIED AND ENVIRONMENTAL MICROBIOLOGY | | | 4.077 |
| 6 | 861 |  | 0.03 | | 1 | | JOURNAL OF PERIODONTOLOGY | | | 2.768 |
| 7 | 854 |  | 0.05 | | 1 | | JOURNAL OF BACTERIOLOGY | | | 3.234 |
| 8 | 835 | 5.07 | 0.03 | | 1 | | INFECTION AND IMMUNITY | | | 3.16 |
| 9 | 804 | 14.3 | 0.01 | | 1 | | ORAL MICROBIOLOGY AND IMMUNOLOGY | | | 0 |
| 10 | 785 |  | 0.05 | | 1 | | PNAS | | | 9.58 |
| 11 | 748 | 17.1 | 0.03 | | 2 | | ARCHIVES OF ORAL BIOLOGY | | | 1.663 |
| 12 | 703 |  | 0.05 | | 1 | | PERIODONTOL 2000 | | | 0 |
| 13 | 694 |  | 0.08 | | 1 | | NATURE | | | 43.07 |
| 14 | 657 |  | 0.01 | | 1 | | ISME JOURNAL | | | 9.493 |
| 15 | 580 |  | 0.05 | | 1 | | SCIENCE | | | 41.037 |
| 16 | 538 | 5.95 | 0.05 | | 1 | | JOURNAL OF PERIODONTAL RESEARCH | | | 2.613 |
| 17 | 532 |  | 0.04 | | 1 | | ORAL DISEASES | | | 2.625 |
| 18 | 531 | 7.99 | 0.05 | | 1 | | CARIES RESEARCH | | | 2.326 |
| 19 | 480 |  | 0 | | 1 | | BMC MICROBIOLOGY | | | 3.287 |
| 20 | 469 |  | 0.04 | | 1 | | NUCLEIC ACIDS RESEARCH | | | 11.147 |
| 21 | 442 |  | 0.01 | | 1 | | NATURE REVIEWS MICROBIOLOGY | | | 34.648 |
| 22 | 441 | 88.7 | 0 | | 1 | | SCIENTIFIC REPORTS | | | 4.011 |
| 23 | 425 |  | 0.08 | | 1 | | JOURNAL OF MEDICAL MICROBIOLOGY | | | 1.926 |
| 24 | 406 | 8.72 | 0.03 | | 1 | | MICROBIOLOGY-SGM | | | 1.922 |
| 25 | 388 |  | 0 | | 1 | | BIOINFORMATICS | | | 1.189 |
| 26 | 375 | 12 | 0 | | 1 | | GENOME BIOLOGY | | | 14.028 |
| 27 | 367 | 17.7 | 0 | | 1 | | NATURE METHODS | | | 28.467 |
| 28 | 366 |  | 0 | | 1 | | MOLECULAR ORAL MICROBIOLOGY | | | 2.925 |
| 29 | 352 |  | 0.03 | | 1 | | GENOME RESEARCH | | | 9.944 |
| 30 | 344 | 36.7 | 0 | | 1 | | JOURNAL OF ORAL MICROBIOLOGY | | | 2.742 |
| 31 | 330 |  | 0.05 | | 1 | | FEMS MICROBIOLOGY LETTERS | | | 1.994 |
| 32 | 328 |  | 0.07 | | 1 | | LANCET | | | 59.102 |
| 33 | 312 | 59 | 0 | | 1 | | FRONTIERS IN MICROBIOLOGY | | | 4.259 |
| 34 | 311 |  | 0.01 | | 1 | | CELL HOST&MICROBE | | | 15.753 |
| 35 | 278 |  | 0.07 | | 1 | | NEW ENGLAND JOURNAL OF MEDICINE | | | 70.67 |
| 36 | 270 | 5.88 | 0.01 | | 1 | | TRENDS IN MICROBIOLOGY | | | 11.974 |
| 37 | 266 | 30.4 | 0.13 | | 37 | | ORAL SURGERY ORAL MEDICINR ORAL PATHOLOGY ORAL RADIOLOGY | | | 1.69 |
| 38 | 252 | 26.5 | 0 | | 1 | | MBIO | | | 6.747 |
| 39 | 244 | 4.22 | 0 | | 1 | | PLOS PATHOGENS | | | 6.463 |
| 40 | 238 | 33.5 | 0.09 | | 18 | | JOURNAL OF THE AMERICAN DENTAL ASSOCIATION | | | 2.572 |
| 41 | 222 | 20.2 | 0.16 | | 19 | | ACTA ODONTOLOGICA SCANDINAVICA | | | 1.565 |
| 42 | 216 | 9.74 | 0.13 | | 3 | | ADVANCES IN DENTAL RESEARCH | | | 0 |
| 43 | 213 | 52.5 | 0 | | 1 | | MICROBIOME | | | 10.465 |
| 44 | 208 | 21.2 | 0 | | 1 | | CELL | | | 36.216 |
| 45 | 205 | 11.2 | 0.01 | | 1 | | ENVIRONMENTAL MICROBIOLOGY | | | 5.147 |
| 46 | 201 | 13.7 | 0.03 | | 2 | | EUROPEAN JOURNAL OF ORAL SCIENCES | | | 1.81 |
| 47 | 188 | 16.1 | 0 | | 1 | | BMC ORAL HEALTH | | | 2.048 |
| 48 | 180 | 40.8 | 0.02 | | 2 | | GUT | | | 17.943 |
| 49 | 160 | 43.5 | 0.02 | | 2 | | CRITICAL REVIEWS IN ORAL BIOLOGY & MEDICINE | | | 0 |
| 50 | 158 | 38.9 | 0 | | 1 | | FRONTIERS IN CELLULAR AND INFECTION MICROBIOLOGY | | | 3.518 |

**Table S6: The top 20 keywords related to oral microbiome publications between 1959 and 2019.**

| **Rank** | **Frequency** | **Keyword** |
| --- | --- | --- |
| 1 | 721 | periodontal disease |
| 2 | 417 | oral microbiome |
| 3 | 375 | oral microbiota |
| 4 | 329 | dental caries |
| 5 | 327 | bacteria |
| 6 | 284 | health |
| 7 | 281 | diversity |
| 8 | 275 | dental plaque |
| 9 | 272 | porphyromonas gingivali |
| 10 | 230 | saliva |
| 11 | 215 | streptococcus mutan |
| 12 | 192 | biofilm |
| 13 | 189 | oral microflora |
| 14 | 160 | children |
| 15 | 153 | infection |
| 16 | 150 | identification |
| 17 | 133 | community |
| 18 | 118 | association |
| 19 | 105 | fusobacterium nucleatum |
| 20 | 99 | gut microbiota |

**Supplementary Figures:**


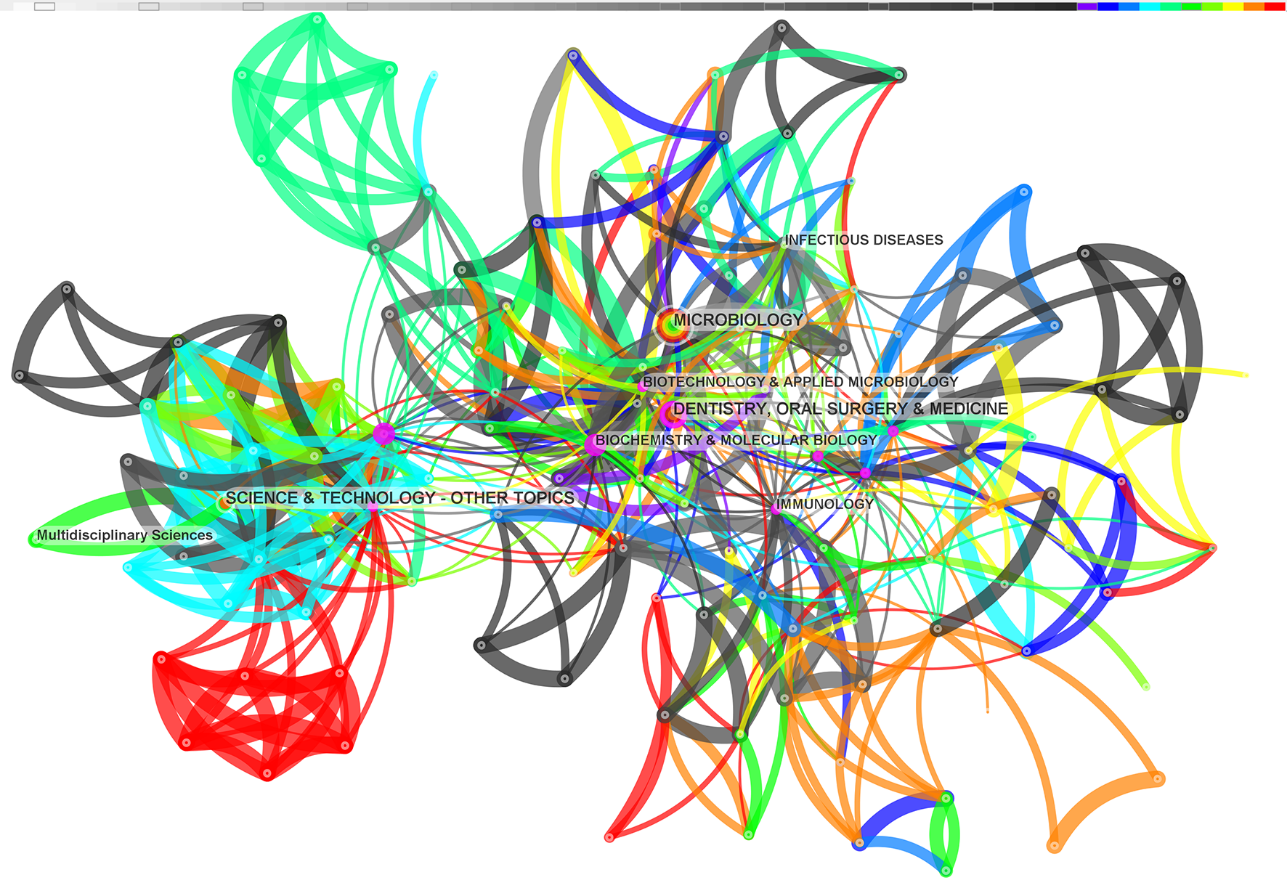


**Figure S1:** **The network of research areas on oral microbiome literatures from 1959 to 2019.** Circular nodes represent areas. The size of a circle is in proportion to the number of literatures on the subject category. The colors of links are corresponding to the year. The purple rims of circles represent the high centralities and the red circles mean the high strength of burst.

**
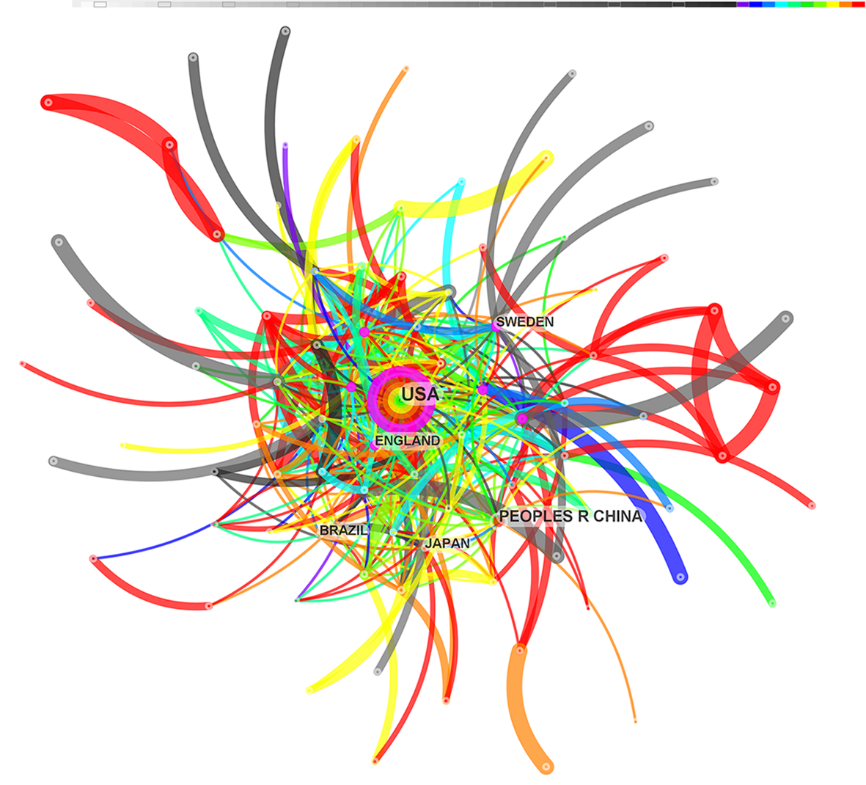
**

**Figure S2: The cooperation map of countries contributed to articles related to the oral microbiome publications between 1959 and 2019.** Circular nodes represent countries. The size of a circle is in proportion to the number of literatures. The colors of links are corresponding to the year. The purple rims of circles represent the high centralities, and the red circles mean the high strength of burst.

**
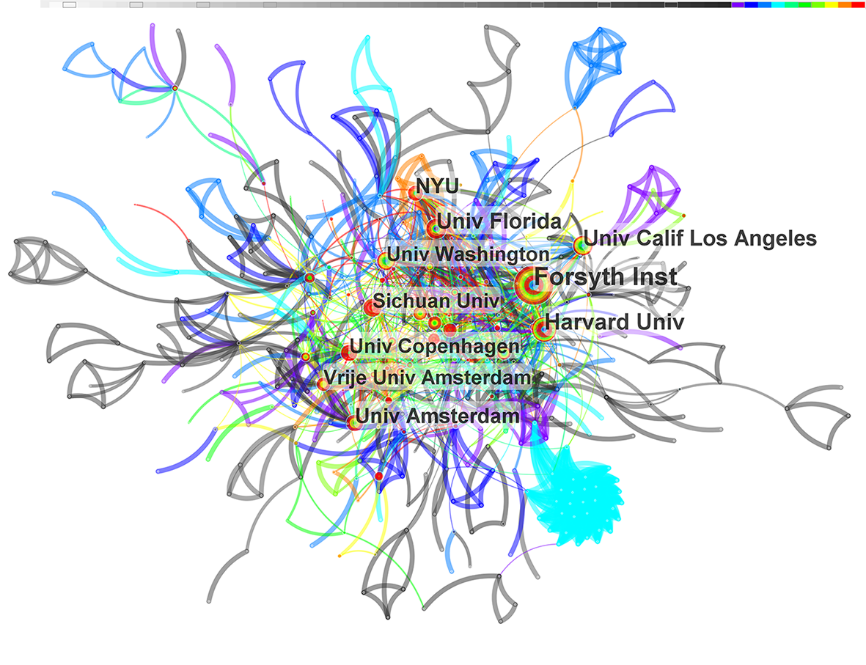
**

**Figure S3: The cooperation map of institutions related to the oral microbiome publications between 1959 and 2019.** The nodes represent institutions, and the size is in proportion to the number of documents. The colors of links are corresponding to the year. The purple rims of circles represent the high centralities, and the red circles mean the high strength of burst.

**
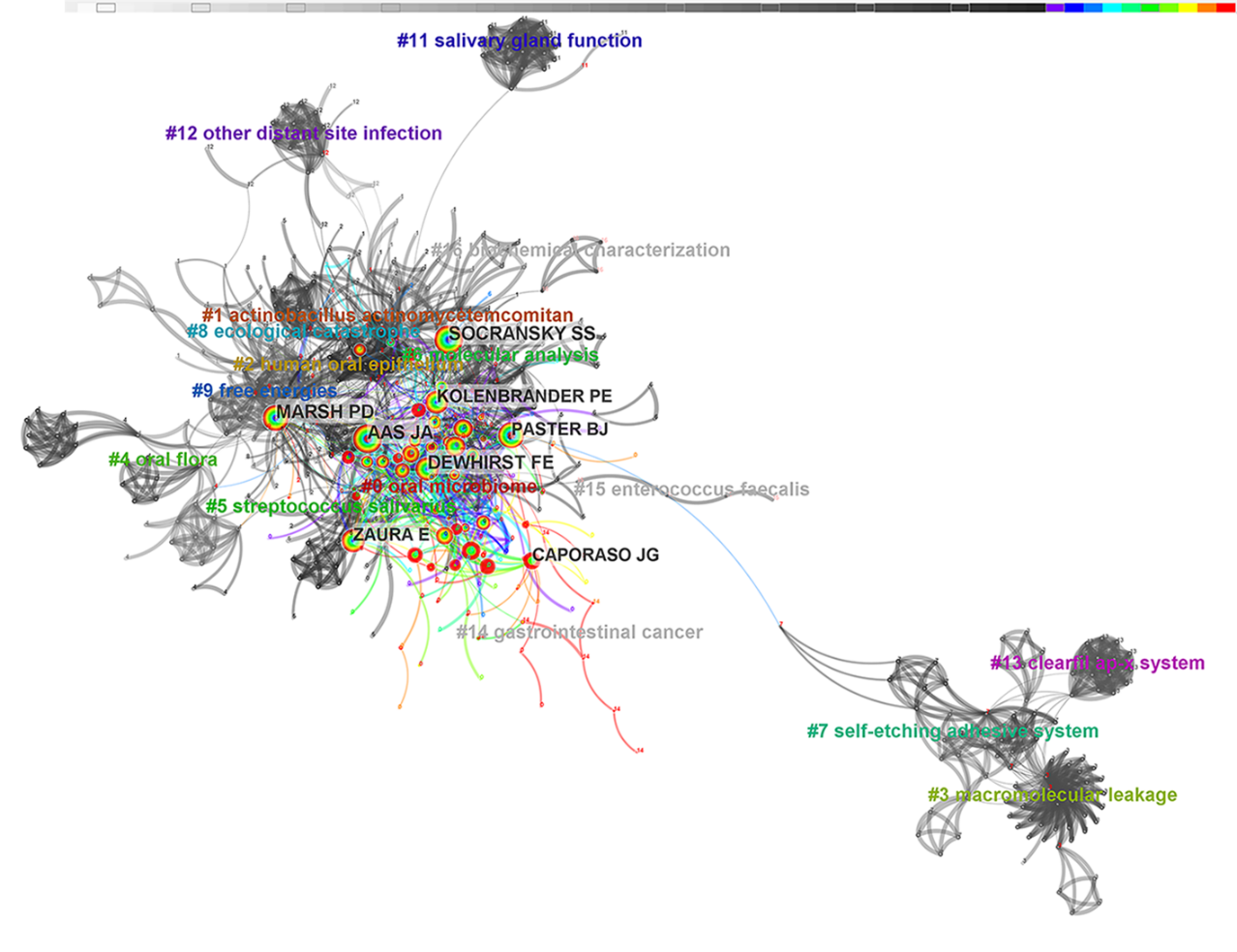
**

**Figure S4: A network of co-cited authors on oral microbiome literatures from 1959 to 2019.** The sequence number of sub-networks and cluster top terms were marked in the central area. Circular nodes represent authors, and the sizes of them are in proportion to the frequencies. The colors of links are corresponding to the year. The purple rims of circles represent the high centralities, and the red circles mean the high strength of bursts.

**
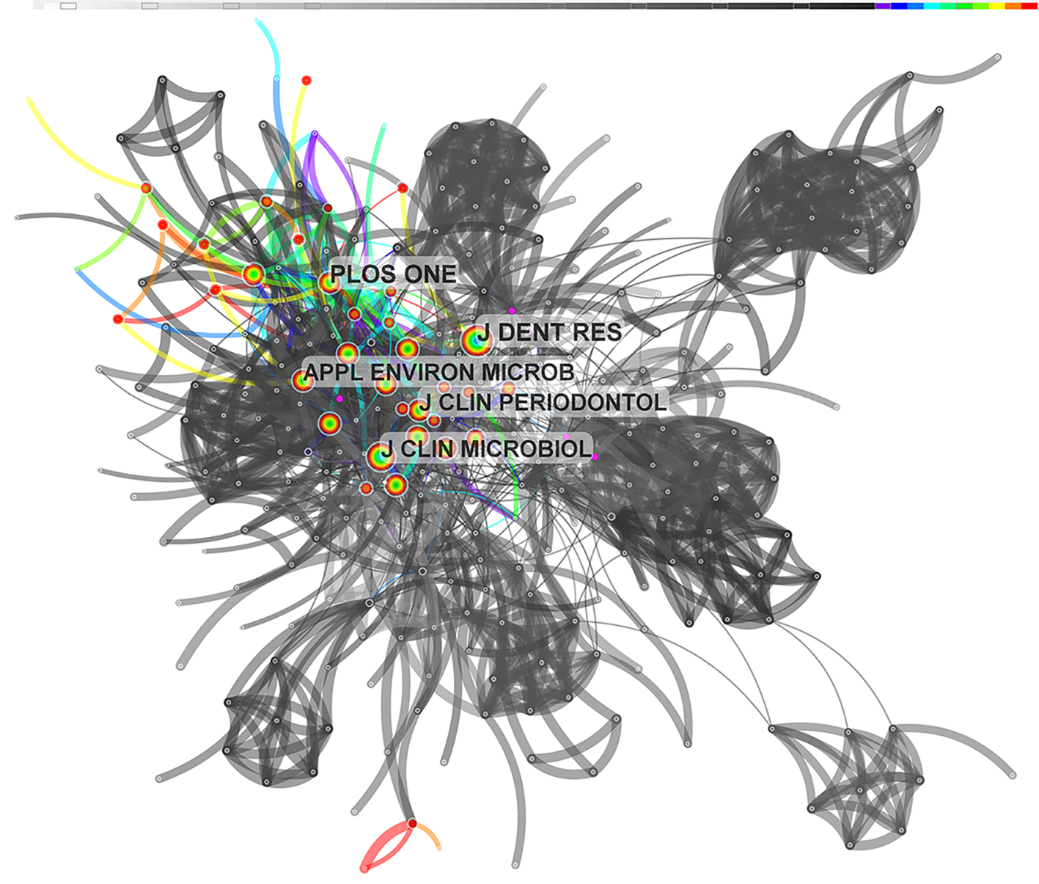
**

**Figure S5:** **The network of co-cited journals related to oral microbiome publications from 1959 to 2019.** Circular nodes represent journals, and the sizes of them are in proportion to the frequencies. The colors of links are corresponding to the year. The purple rims of circles represent the high centralities, and the red circles mean the high strength of bursts.


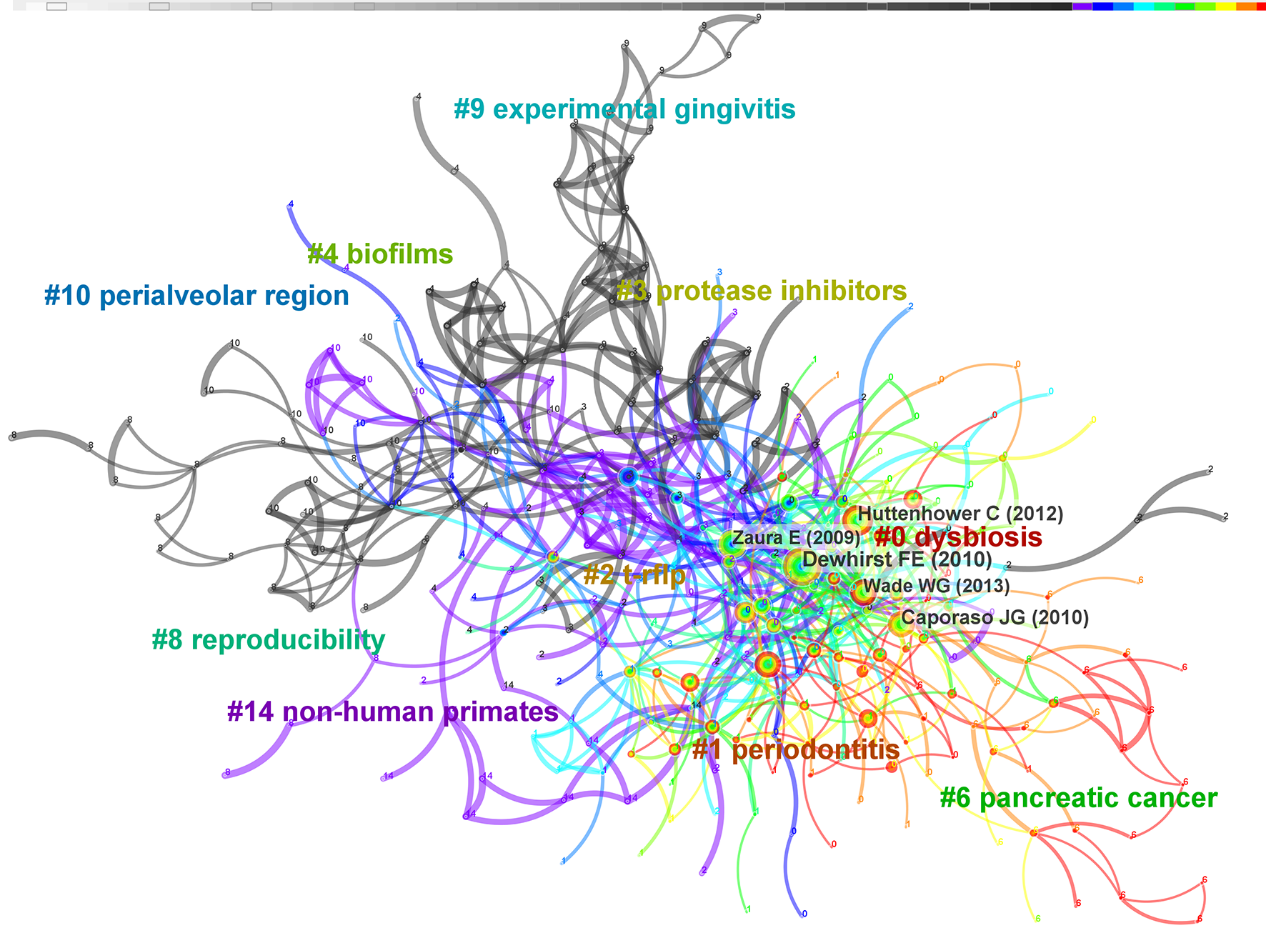


**Figure S6: A visual clustering network of co-citation references related to oral microbiome literatures between 1959 and 2019.** According to the parameters above, 629 cited-references structured the network and there were 10 main clusters. The sequence number of sub-networks and cluster top terms were marked in the central area. The colors of labels from warm to cold are in accordance with the sizes of clusters. Clusters are referred to in terms of the labels selected by the log-likelihood ratio test method (LLR).
